# Efficiency Enhancement of a Cantilever-Based Vibration Energy Harvester

**DOI:** 10.3390/s140100188

**Published:** 2013-12-23

**Authors:** Ali E. Kubba, Kyle Jiang

**Affiliations:** 1 Fusion Innovations Ltd., Research and Innovation Services, Birmingham Research Park, Vincent Drive, Edgbaston, Birmingham, B15 2SQ, UK; 2 School of Mechanical Engineering, University of Birmingham, Edgbaston, Birmingham, B15 2TT, UK; E-Mail: k.jiang@bham.ac.uk

**Keywords:** energy harvesting, TPMS, piezoceramic, vibration, harmonic excitation energy, damping, FEA

## Abstract

Extracting energy from ambient vibration to power wireless sensor nodes has been an attractive area of research, particularly in the automotive monitoring field. This article reports the design, analysis and testing of a vibration energy harvesting device based on a miniature asymmetric air-spaced cantilever. The developed design offers high power density, and delivers electric power that is sufficient to support most wireless sensor nodes for structural health monitoring (SHM) applications. The optimized design underwent three evolutionary steps, starting from a simple cantilever design, going through an air-spaced cantilever, and ending up with an optimized air-spaced geometry with boosted power density level. Finite Element Analysis (FEA) was used as an initial tool to compare the three geometries' stiffness (K), output open-circuit voltage (V_ave_), and average normal strain in the piezoelectric transducer (ε_ave_) that directly affect its output voltage. Experimental tests were also carried out in order to examine the energy harvesting level in each of the three designs. The experimental results show how to boost the power output level in a thin air-spaced cantilever beam for energy within the same space envelope. The developed thin air-spaced cantilever (8.37 cm^3^), has a maximum power output of 2.05 mW (H = 29.29 μJ/cycle).

## 1. Introduction

Most of the energy harvesting units found in the literature are based on vibration using piezoelectric transducers. This is attributed to the simplicity of these systems and the level of energy offered by vibration sources [1,2] and also because of the piezoelectric compatibility with electronic devices, particularly commercial portable devices and wireless sensor nodes [3]. In this research, for the purpose of energy harvesting from a rolling tyre, vibration-based energy harvesting is an option [4–12].

Tyre vibration is an attractive energy source in which energy harvesting might be applied. Several studies have been completed to measure tyre vibration under different loading and road surface conditions using different techniques. For instance, the Pirelli Tire System project in co-operation with the Mechanical Engineering Department of the Politecnico di Milano have published a paper regarding measurements of pneumatic tyre acceleration under rolling conditions using a three-axial MEMS accelerometer [13]. From this paper, it can be seen that harvestable vibration energy is around the 100 Hz range. Kindt *et al.* [14] carried out experiments on tyre vibration, and collected experimental data using a Laser Doppler vibrometer and the high power vibration energy density was also around 100 Hz. A similar frequency spectrum pattern was obtained by Roundy [15,16] and Löhndorf *et al.* [6]. Vibration based piezoelectric, electrostatic and electromagnetic micro generators for tyre pressure monitoring have been developed by several researchers and companies [9–11,17–24], but in most cases, micro generator performance highly depends on the applied frequency in such a way that it has a quite narrow band width of the efficient power generation level around its resonance frequency which makes it not suitable for the variable excitation frequency environment, such as in land vehicle tyres. However, vibration energy harvesters can be a good option when applied on constant speed machinery by toning their resonance frequencies with the machines' operation speeds. Khameneifar and Arzanpour [5] made a theoretical model for a bending-based energy harvester attached on a pneumatic inner tyre surface in which the generated electric charge was proportional to tyre speed and radial deflection. Calculation findings can be summarised to a prediction of a power generation of approximately 2.95 mW at 50 km/h when a 30 kΩ load resistor is used. Its also worth mentioning that tyre induced vibration is highly affected by road surface roughness which can change vibration velocity and acceleration amplitudes [13,25]. This can directly affect the amount of the harvested energy when a vibration energy harvester is employed.

Active TPMSs power consumes less than passive TPMSs, with a power consumption around 200–250 μWs for a State-of-the-Art TPMS module [26]. The maximum power output level of vibration energy harvester in the literature found to be between 2.5 μW and 349 μW and as shown in Table 1.

This article presents the design, theoretical analysis, FEA simulations and experimental investigation of a thin piezoelectric based vibration energy harvester. The DuraAct patch transducer element (DPT) used in this study is a compact and flexible unit which utilizes a thin piezoceramic foil sandwiched between two conductive films all embedded in a ductile composite-polymer structure, labelled (DuraAct P-876.A11) and developed by PI (Physik Instrumente) Ltd. The performance of the developed design differs from conventional cantilever based energy harvesters in terms of the output power level and density. Following the energy harvester analysis, a power management circuit designed by the author is also presented. Finally the developed power management circuit is employed to link between the promoted energy harvester and a capacitive sensor readout circuit designed by the author.

## 2. Characterizing the DPT Element

**Figure 1.**
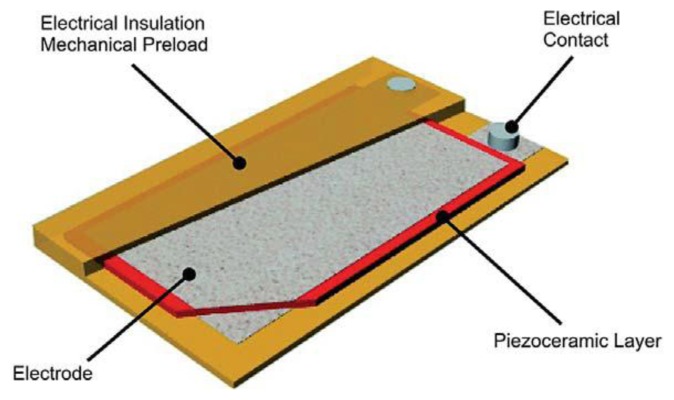
Design principle of the DPT transducer. Published courtesy of PI Ltd.

**Figure 2.**
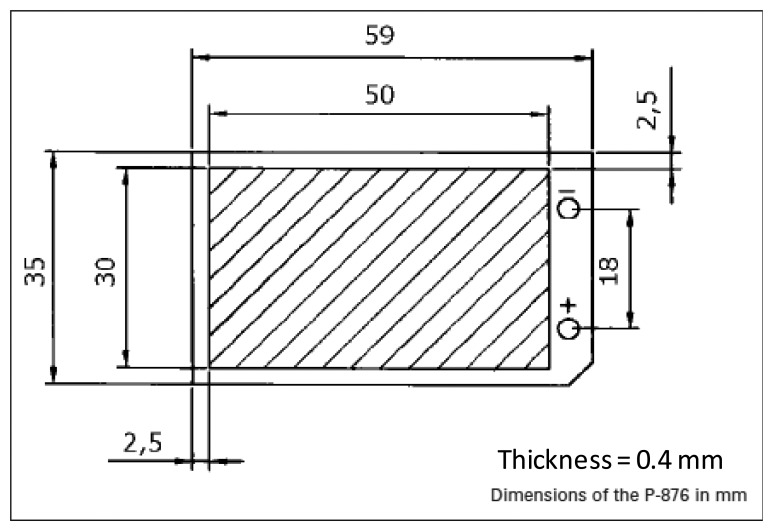
Dimensions of the P-876. A11 piezoelectric patch transducer. Published courtesy of PI Ltd.

This section presents the main features of the DPT transducer used in this study (see Figure 1). The reason behind choosing this transducer is its high charge coefficient *d*_33_ (394 pC/N), high mechanical strength and flexibility, and wide operation temperature range (−20 to 150 °C), making it well suited for energy harvesting within a tyre environment. The main dimensions of the transducer are shown in Figure 2.

In addition to the electrical and mechanical characteristics of the transducer given by the manufacturer, experimental tests were carried out in the school of mechanical engineering laboratories to find out more about the mechanical and electrical properties this transducer.

**Figure 3.**
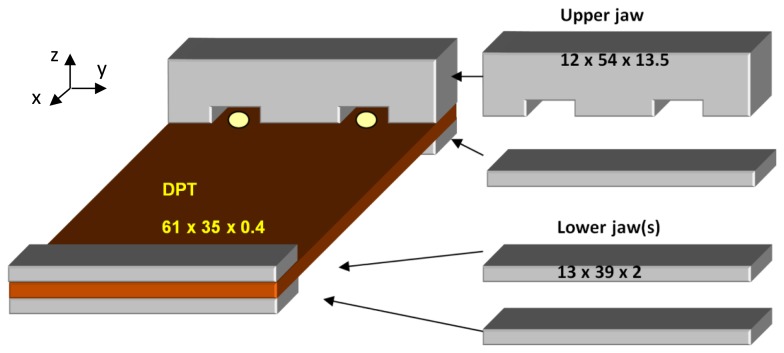
Mounting configuration of the DPT transducer in the experimental tests.

**Figure 4.**
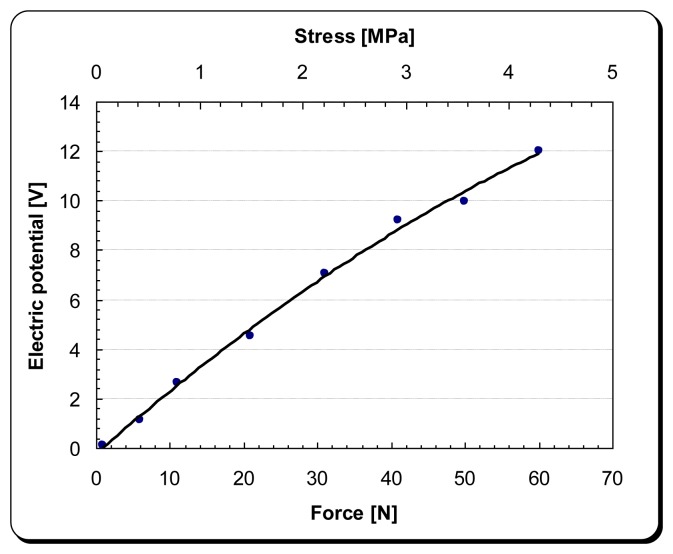
Force *vs.* open circuit electric potential curve of the DPT transducer [P-876 DuraAct™ (P-876.A11)].

**Figure 5.**
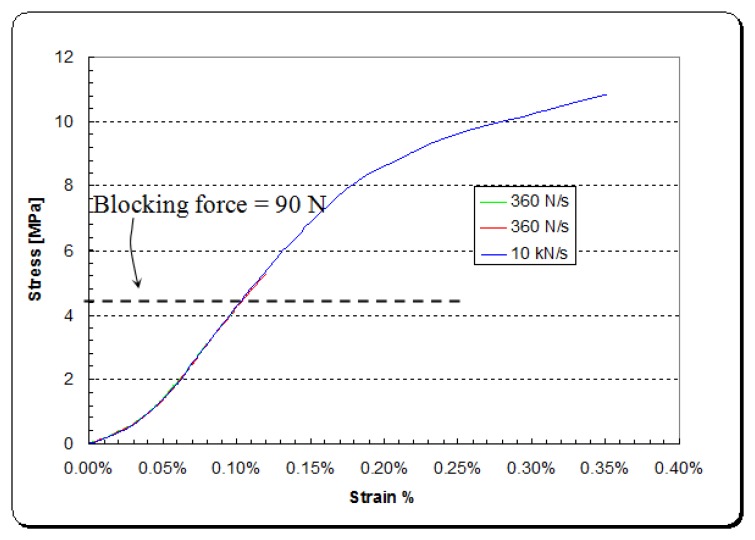
Tensile stress-strain curve of the DPT transducer [P-876 DuraAct™ (P-876.A11)].

In order not to damage the electrical contacts of the DPT transducer, a couple of custom made jaws were used as shown in Figure 3. The experimental tests which were carried out examined the piezoelectric effect and Young's modulus of elasticity of the transducer at different force-rates along the x-axis, as shown in Figures 4 and 5, respectively.

**Figure 6.**
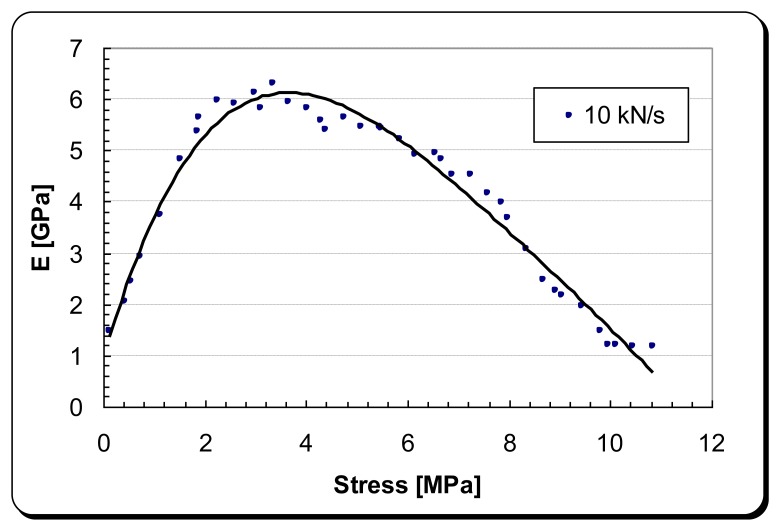
Tensile modulus elasticity of the DPT transducer [P-876 DuraAct™ (P-876.A11)] as a function of tensile force.

Figure 5 shows that the DPT transducer has a hyperelastic stress-strain relationship in the x-direction. This results in a variable modulus of elasticity for the DPT transducer, depending on how much tensile stress is being applied as shown in Figure 6. Modulus of elasticity experimental data were fed into the FEA modelling.

Non-linearity is a fundamental behavior in piezoelectric materials [30]. Figure 5 shows non-linearity in the stress-strain curve. This could be also due to the laminated structure of the patch transducer which consists of a piezoceramic plate, electrodes and preloaded polymer materials acting as mechanical preload and also as electrical insulation, making the DuraAct bendable [31]. The blocking force can be defined as the force required for pushing back a fully energized actuator to zero displacement.

**Figure 7.**
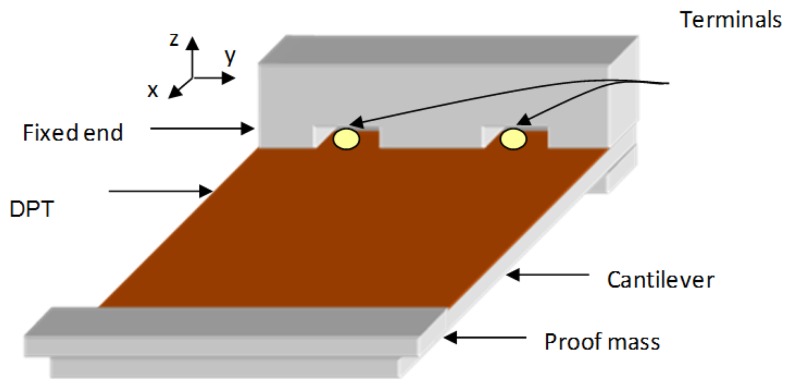
Vibration based energy harvester configuration.

The next section presents the development of a high power density energy harvester through modifying the design of the cantilever that holds the DPT transducer as shown in Figure 7.

## 3. Cantilever Design Optimization

Cantilever geometry in a vibration based energy harvester is a crucial factor to the efficiency and effectiveness of the device. Having electric charge generated by a piezoelectric element is mainly influenced by the strain distribution within the transducer. Optimized cantilever designs usually tend to increase the average strain value across the transducer surface area and prevent overstraining the transducer [32–34]. Zheng [35,36] presents an alternative cantilever design; that is an air spaced cantilever, in which a fairly even strain distribution across the piezoelectric transducer layer is obtained by increasing the distance between the piezoelectric transducer layer and the neutral plane of bending [35].

**Figure 8.**
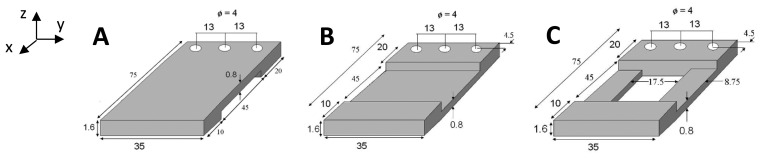
Configuration of the three cantilevers (all dimensions are in mm).

Figure 8 shows the three evolutionary steps, (designs A, B and C), which the optimized design underwent; starting from a simple cantilever design, going through an air-spaced cantilever, and ending up with an optimized air-spaced geometry with a boosted power density level. The same proof mass (47 g) is used in all designs, and all geometries are contained in the same space envelope (see Figure 7). Dimensions were chosen to achieve high deflection in the DFT but also within the fatigue limits of the cantilever assembly.

**Figure 9.**
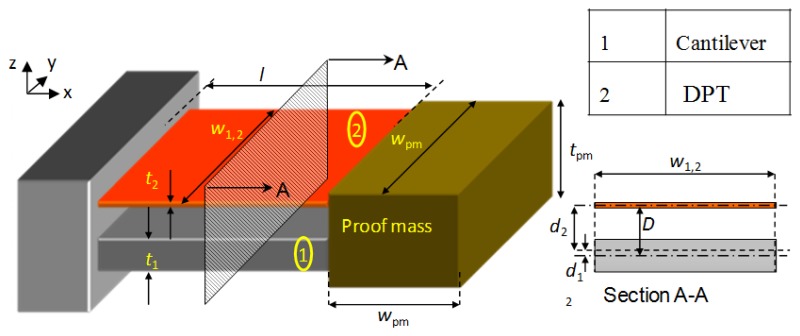
Schematic diagram of the vibration-based energy harvester.

**Figure 10.**
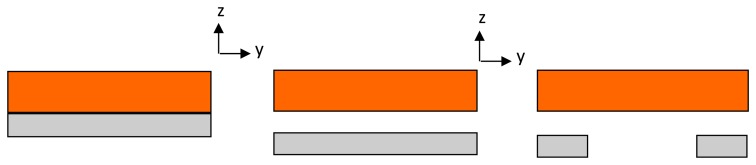
Section A-A of the energy harvester for the three cantilever designs.

In order to compare between the three designs' performances, properties like the flexural rigidity and the average normal strain distribution in the x-direction (ε_x_)_avg_ need to be examined. In the following paragraphs, analytical calculations, using the formulae derived by Zheng [35], and FEA simulations using COMSOL software for all designs, are presented. A general schematic diagram for the vibration-based energy harvester is shown in Figure 9 below. Cross-sections of the energy harvester for the three cantilever designs are shown in Figure 10.

### 3.1. Theoretical Analysis

This section presents the theoretical formulae and the corresponding calculated values of the fundamental parameters of the energy harvester assembly for the three cantilever designs. The formula used in this analysis presented in Table 2 are quoted from Zheng and Xu [35]. Table 3 shows the given data and calculated mechanical and electrical parameters needed to compare between the influences of each of the three cantilever designs in the performance of the vibration-based energy harvester.

In order to determine the location of the neutral axis for the cantilever-DPT assemblies shown in Figure 10, the following formula is applied [37]:

(1) $$E_{1}\int_{1}\text{ydA}-E_{2}\int_{2}\text{ydA}=0$$

where the variables are defined in Table 3.

**Figure 11.**
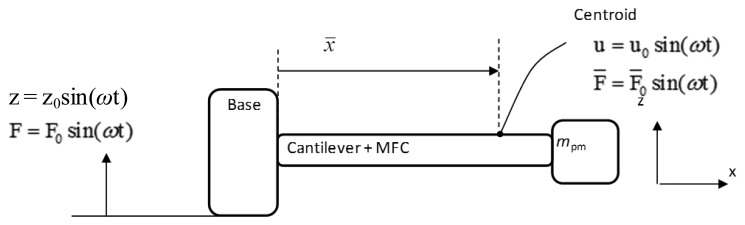
Harmonic excitation of the vibration-based energy harvester.

Having the energy harvester harmonically excited by the motion of the supported points as shown in Figure 11, the energy balance of the energy harvesting system from time *t*_i_ to *t*_f_ can be obtained [38,39]:

(2) $$\int_{t_{i}}^{t_{f}}F(t)\dot{z}(t)dt=\int_{t_{i}}^{t_{f}}c{\dot{z}}^{2}(t)dt+\int_{t_{i}}^{t_{f}}V_{P}(t)I(t)dt$$

where *F* is the harmonic excitation force in N, ż is the excitation speed in m/s, *c* is the damping coefficient in N s/m, *V*_P_ is the voltage across the piezoelectric element in V, and *I* is the output current generated by the piezoelectric element in A.

The term on the left-hand side represents the input mechanical energy (*E*_inp_). The first term on the right-hand side represents the energy dissipated due to mechanical damping within the cantilever-DPT assembly (*E*_dsp_). According to Shu and Lien [38], and assuming a 90° phase difference (θ = 90°), these two terms can be re-written as:

(3) $$\begin{array}{ll} E_{\text{inp}} & =\int_{t_{i}}^{t_{f}}F(t)\dot{z}(t)dt=\frac{\pi }{2}F_{0}z_{0}\\ E_{\text{dsp}} & =\int_{t_{i}}^{t_{f}}c{\dot{z}}^{2}(t)dt=\frac{\pi }{2}\eta_{m}\omega {z_{0}}^{2} \end{array}$$

where *t*_f_–*t_i_* equals $\frac{\pi }{\omega }$ (one half of an oscillation cycle), *F*_0_ is the magnitude of the harmonic excitation force (*F*_0_sin*ωt*) in N, *z*_0_ is the excitation amplitude in m, and *ω* is the excitation frequency in rad/s.

**Figure 12.**
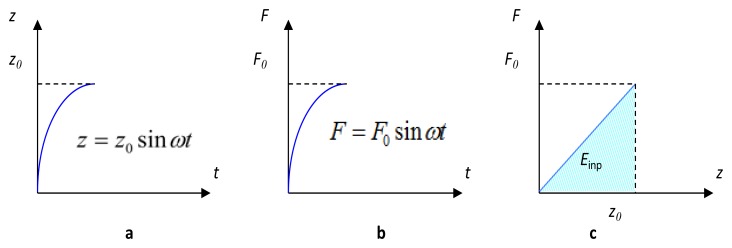
Harmonic waveforms of one quarter of an oscillation cycle of (**a**) displacement and (**b**) force; (**c**) Harmonic force *versus* displacement.

Although both force and displacement experienced by the oscillating mass are harmonic, the relationship between them is still linear as shown in Figure 12.

Thereby, the input mechanical energy for a quarter oscillation cycle can be obtained as:

(4) $$E_{\text{inp}}=\frac{1}{2}F_{0}z_{0}$$

Consequently, the total input mechanical energy for a complete oscillation cycle would be:

(5) $$E_{\text{inp}}=2F_{0}z_{0}$$

Similarly, the energy dissipated due to mechanical damping can be written as:

(6) $$E_{\text{dsp}}=2c\dot{z}z_{0}=2c\omega {z_{0}}^{2}$$

**Figure 13.**
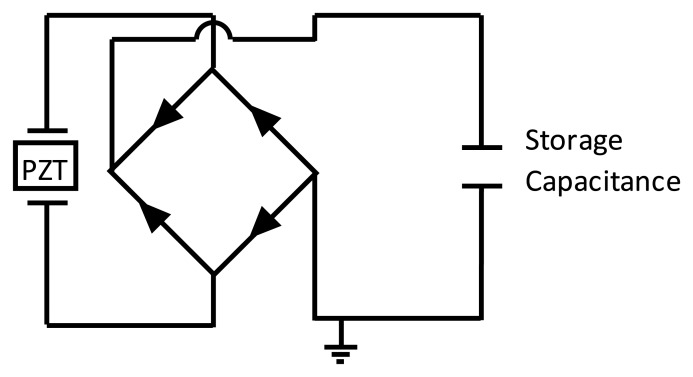
Schematic diagram for the energy storing circuit.

The second term on the right-hand side in Equation (2) represents the electrical energy generated by the energy harvester (*E*_gen_). In this study, the electrical energy generated by the harvester is stored in a 2,200 μF storage capacitor (*C*) after passing through a full bridge rectifier as shown in Figure 13. By neglecting any rectification losses and any other electrical losses, the energy stored in the storage capacitor (*E*_out_) can be assumed to be equal to the energy generated by the energy harvester (*E*_out_ = *E*_gen_) and can be obtained as follows [40]:

(7) $$E_{\text{out}}=\frac{1}{2}({V_{f}}^{2}-{V_{i}}^{2})$$

where *V*_i_ and *V*_f_ are the stored voltage in the storage capacitor at *t*_i_ to *t*_f_, respectively.

The overall system efficiency can be obtained as:

(8) $$\eta =\frac{E_{\text{out}}}{E_{\text{inp}}}$$

In this analysis, the cantilever has a relatively considerable mass *m*_c_ compared to the proof mass *m*_pm_. For this reason it is assumed that the calculations for the kinetic energy, harmonic excitation force, and the amplitude of oscillation of the vibrating energy harvester consider the overall oscillating mass (*m*_c_ + *m*_pm_ + *m_dpt_*) and at the centroid of the total oscillating mass, which is calculated using the first moment of mass as presented in Table 3 [37]. Locations of the centre of mass for the three designs are listed in Table 3. The periodic motion of the cantilever-DPT assembly is assumed to follow the harmonic motion formulae as follows [41]:

(9) $$u=u_{0}\sin\omega t$$

where *u*_0_ is the amplitude of oscillation at position of the centroid in m, and *t* is time in s.

Velocity and acceleration of oscillation are the first and second time derivatives of Equation (9) respectively [41]:

(10) $$\dot{u}=\omega u_{0}\sin(\omega t\frac{\pi }{2})$$

(11) $$\ddot{u}=\omega^{2}u_{0}\sin(\omega t+\pi )=-\omega^{2}u_{0}\sin\omega t$$

**Figure 14.**
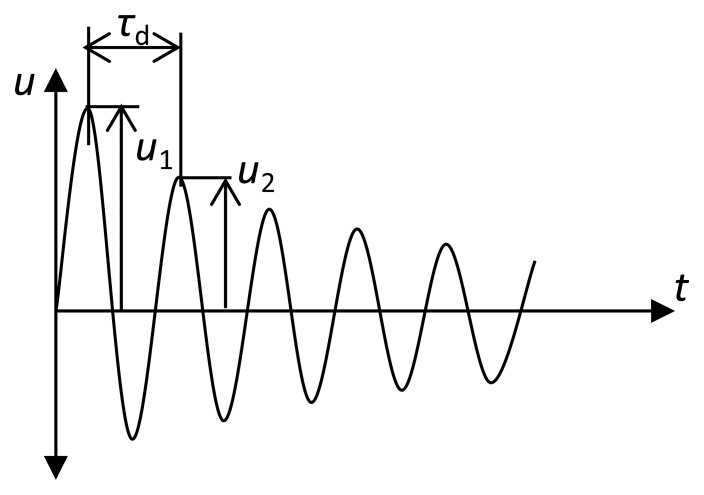
Rate of decay of oscillation measured by logarithmic decrement [41].

The damping coefficient *c* is measured experimentally, firstly by observing the rate of decay *ξ* under free vibration (see Figure 14) and by using the logarithmic decrement formula [41]:

(12) $$\delta =\ln\frac{u_{1}}{u_{2}}=\xi \omega_{n}\tau_{d}$$

where *δ* is the logarithmic decrement coefficient, *u*_1_ and *u*_2_ are the amplitudes of two neighboring cycles in m, *ω*_n_ is the resonance frequency in rad/s, and *τ*_d_ is the damped period of oscillation in s.

Then the damping coefficient *c* can be obtained using the following formula [41]:

(13) $$c=2\xi \omega_{n}m$$

where *m* is the total oscillating mass in kg.

**Figure 15.**
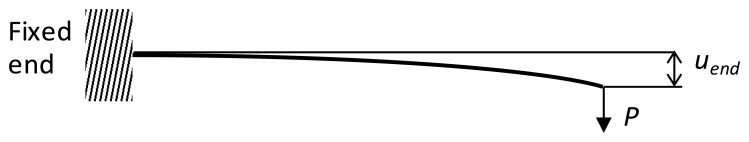
Cantilever stiffness test loading configuration.

The amplitude at resonance can be found to be [41]:

(14) $$u_{0}=\frac{{\bar{F}}_{0}}{2\xi k}$$

where *F̅*_0_ is the magnitude of the induced force (*F̅*_0_ sin *ωt*) at position of centroid in N, *k* is the cantilever-DPT assembly stiffness at position of load *P* (see Figure 15) in N/m.

Using Newton's second law, the induced force at the position of centroid can be obtained:

(15) $$\bar{F}=m\ddot{u}=-\omega^{2}u_{0}\sin\omega t$$

Using Equation (5), Equation (15) can be rewritten as:

(16) $$E_{\text{inp}}=2{\bar{F}}_{0}u_{0}$$

Similarly, the energy dissipated due to mechanical damping can be written as:

(17) $$E_{\text{dsp}}=2c\omega {u_{0}}^{2}$$

By substituting Equations (16) and (17) into Equation (2), the energy balance equation can be rewritten as:

(18) $$E_{\text{inp}}=2{\bar{F}}_{0}u_{0}=2c\omega {u_{0}}^{2}+E_{\text{out}}$$

Having only the mechanical damping influence considered in the energy balance equation as the main source of energy dissipation, a fourth term (*E*_los_) is added to contain any other losses within the system, e.g., electrically induced damping. Therefore, Equation (18) can be rewritten as:

(19) $$E_{\text{inp}}=2{\bar{F}}_{0}u_{0}=2c\omega {u_{0}}^{2}+E_{\text{out}}+E_{\text{los}}$$

The following section presents preliminary numerical results of the characteristics of energy harvesters obtained using COMSOL software.

### 3.2. Numerical Analysis

This section presents the FEA simulations for the three cantilever-DPT assembly designs in terms of mechanical properties; e.g., deflection, resonance frequency and normal strain across the DPT element, and electrical response; that is the voltage generated by the DPT element. The FEA software used to carryout the numerical analysis for the vibration-based energy is COMSOL.

Settings and limitations of the FEA can be summarized by:

- Isotropic mechanical properties for both the cantilever and the DPT element.
- Uniform gap between the cantilever and the DPT element in the harvester assembly for each case study.
- Stress-free condition when no load is applied to the harvester assembly.

**Figure 16.**
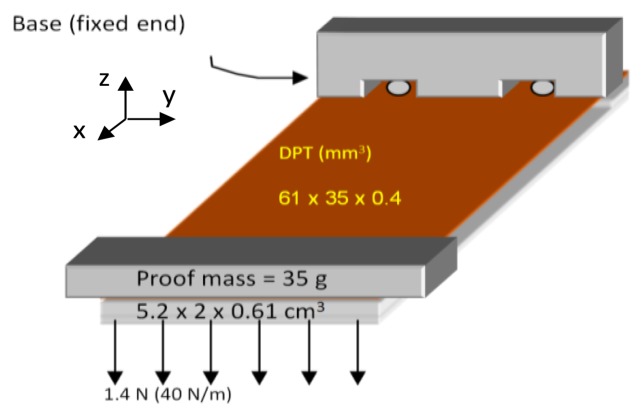
Schematics of the vibration energy harvester and FEA simulation boundary conditions.

Simulation was carried out in two categories: static and modal analysis. The former includes the cantilever-DPT assembly stiffness and the voltage output of the harvester (Figure 16). The latter determines the cantilever-DPT natural frequency. The three cantilever designs were considered in both analyses. A summary of the main results found in these simulations are shown in Table 4.

From Table 4 it can be seen that design C is the best among the three designs for energy harvesting purposes as it offers the highest normal strain (*ε*_avg_) within the DPT layer, and therefore generates the highest electrical charge under the same loading conditions.

**Figure 17.**
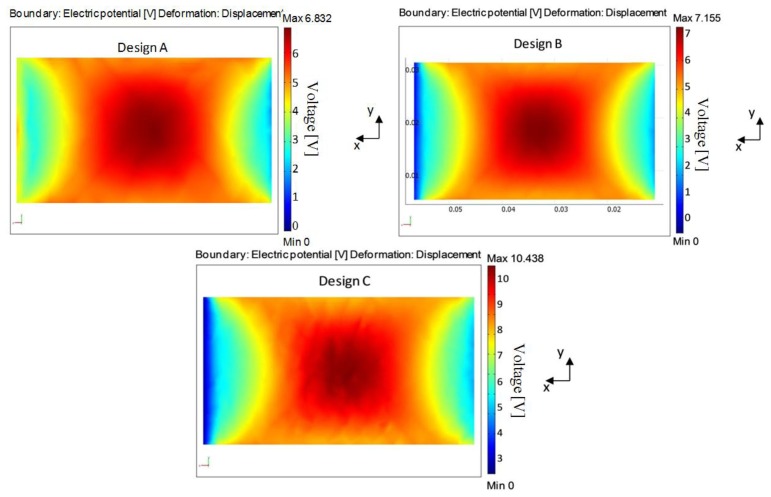
Voltage distribution developed in the DPT transducer under the boundary conditions shown in Figure 16.

Figure 17 below shows the voltage output of the piezoelectric layer when the cantilever-DPT assembly is subjected to a vertical static load of 1.4 N at the free end as shown in Figure 16. Higher voltage values can be observed in design C.

## 4. Experimental

**Figure 18.**
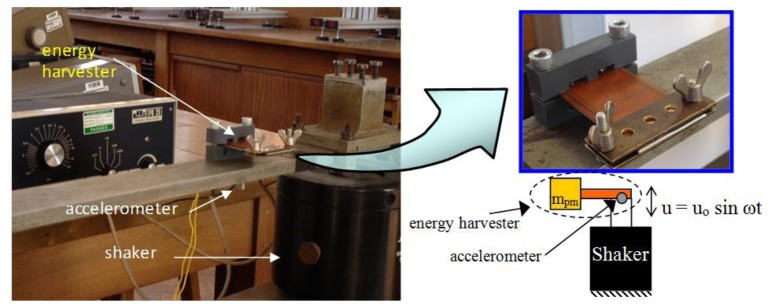
Experimental apparatus and a schematic diagram for testing the energy harvester performance.

This section presents the experimental procedures and the obtained results of testing the vibration energy harvester for the three different cantilever designs. Obtained results include rate of decay of oscillation, cantilever-DPT assembly stiffness, resonance frequency, acceleration of oscillation, harvested energy and the output power of the energy harvester as a function of the storage capacitor voltage and excitation frequency. The apparatus used are shown in Figure 18. The accelerometer used to measure the acceleration of excitation is a product of Brüel & Kjær (Sound and Vibration Measurement A/S), type 4344 307768.

**Figure 19.**
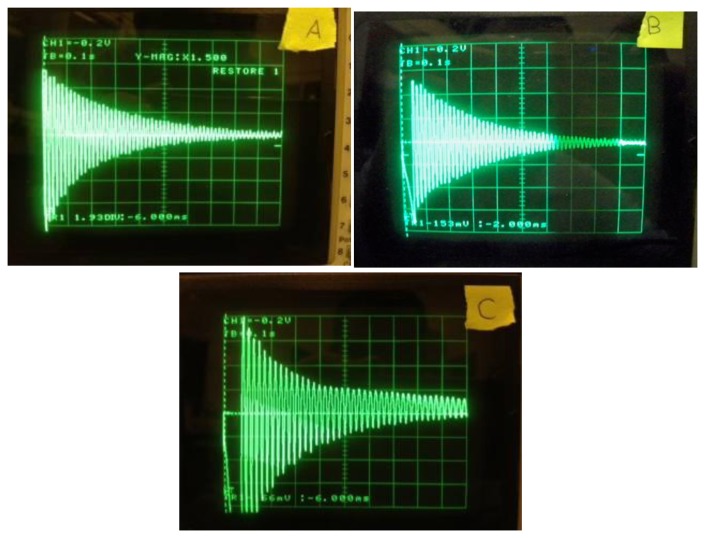
Rate of decay of oscillation measured by the harvester output signal when subjected to free vibration for the three cantilever designs.

Figure 19 shows the collected experimental data of the rate of decay of oscillation for the three cantilever-DPT assemblies.

**Figure 20.**
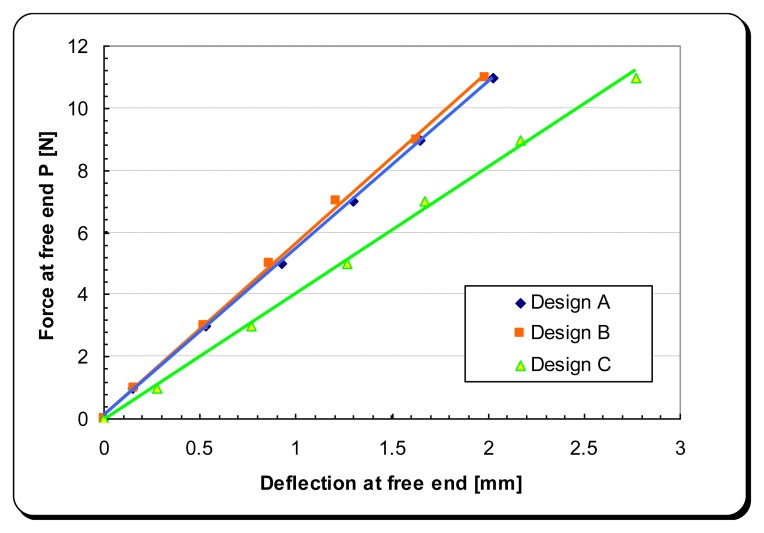
Stiffness test results for the cantilever-DPT assembly for the loading condition shown in Figure 15.

The experimental data of the force *versus* free-end displacement of the three cantilever-DPT assemblies are shown in Figure 20.

Following this, forced vibration tests for the three designs were carried out in two ways:

- Identical excitation acceleration of 0.5 g;
- Identical amplitude of excitation of 40 μm.

**Figure 21.**
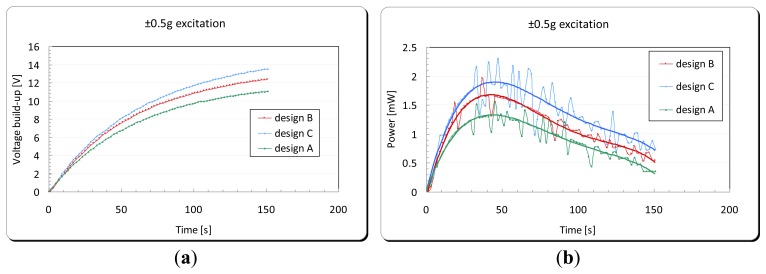
(**a**) Voltage build-up in a 2,200 μF capacitor and (**b**) the corresponding generated power by the energy harvester under ±0.5 g acceleration of excitation.

**Figure 22.**
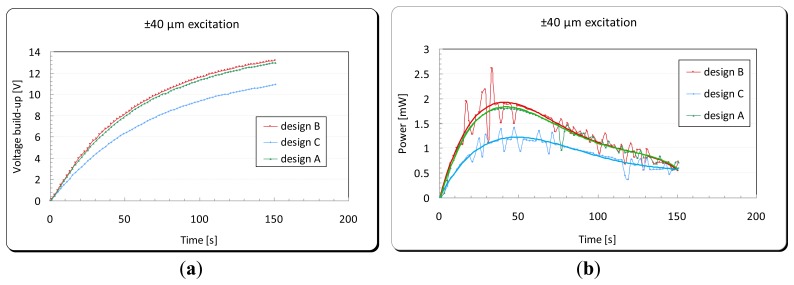
(**a**) Voltage build-up in a 2,200 μF capacitor and (**b**) the corresponding generated power by the energy harvester under ±40 μm amplitude of excitation.

**Figure 23.**
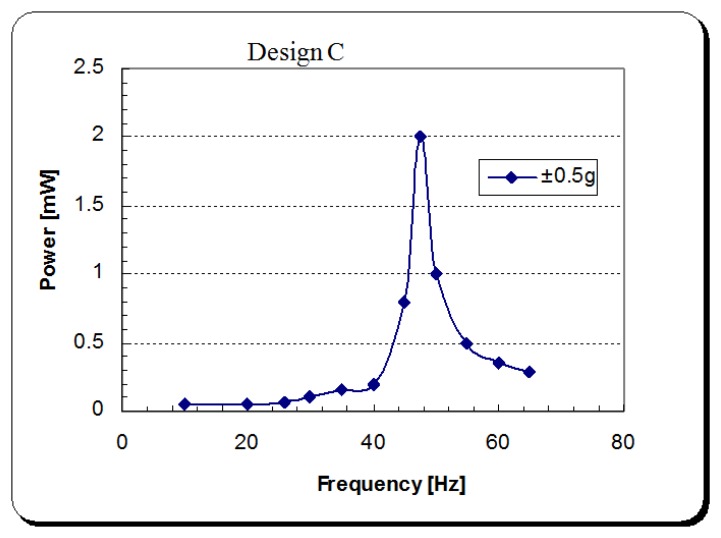
Output power spectrum of the energy harvester under ±0.5 g acceleration of excitation.

Experimental tests, using the apparatus shown in Figure 18, were carried out in order to examine the energy harvesting level obtained in each of the three designs. A full wave rectifier was used in order to convert the generated energy from the DPT from an AC to DC signal. This energy was subsequently stored in a 2,200 μF capacitor (see Figure 13). Each design was excited from the cantilever base by its fundamental structural resonance frequency first with a peak acceleration of ±0.5 g, and then with a ±40 μm amplitude of excitation. The voltage built-up in the storage capacitor and the corresponding generated power by the energy harvester are shown in Figures 21 and 22, respectively. For the energy harvester containing the design C cantilever and undergoing ±0.5 g acceleration of excitation, the output power as a function of excitation frequency is shown in Figure 23.

Table 5 shows a summary of the experimental results for the tests presented above. It also shows the values of the energy main parameters of the harvesting system calculated using the mathematical formulae presented in Section 3.1. The volume power density (*P*_dns_) was calculated using the space envelope containing the energy harvester (6.5 × 5.2 × 0.5) = 16.9 cm^3^.

When comparing the energy generated per one cycle (*H*_out_) and (*P*_avg_) in each cantilever under ±40 μm amplitude of excitation, although design B has the maximum power output among the three designs, it can be seen that there is a dramatic increase in the device efficiency in design C. That is due to the different frequency of oscillation and therefore the time spent to generate that amount of energy. This makes design C the most suitable design among the three studied designs for energy harvesting purposes. The following section presents the integration of the optimized vibration based energy harvester into a developed capacitive sensor read-out circuit designed by the author.

## 5. Power Management Circuitry

**Figure 24.**
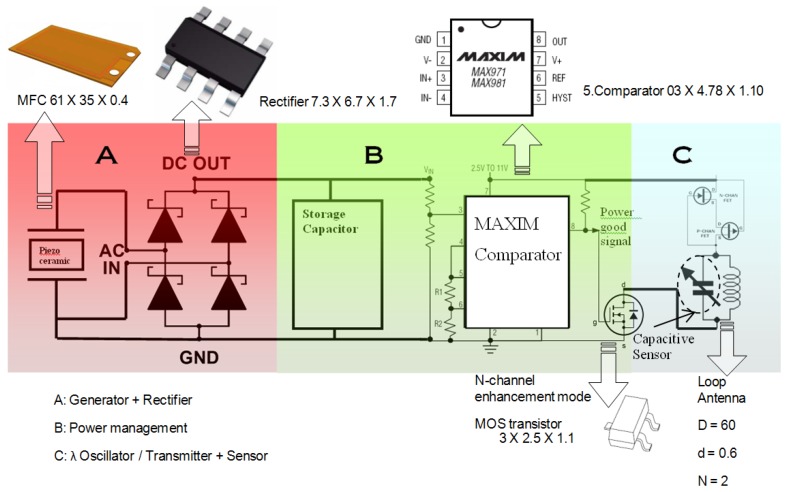
Schematic of the wireless sensor node circuitry.

This section illustrates a power management unit, designed by the author, to regulate the energy generated by the developed vibration-based energy harvester. It also demonstrates the integration of the energy harvester with the power management unit into the read-out circuit presented in this article. A block diagram showing all the three integrated units to make a complete self-powered wireless sensor node powered by vibration energy harvesting is shown in Figure 24.

The power management design requirement is monitor the voltage across the storage capacitor and switching on and off the application load, which is in this case the capacitive sensor read-out and transmission circuit. MAX981 comparator, which is the main component in the power management circuit, was chosen for its low power consumption and therefore minimizing the overall power loss for running the transmission circuit. MAX971/981 comparator acts as the main switch to turn on and off the application circuit (λ oscillator), and therefore it controls the discharge of the storage capacitor whenever the voltage of the capacitor reaches a certain level [42]. The comparator allows switching with hysteresis; that is the turn-on and turn-off voltages are slightly different. This way the system will have an operation voltage range for the application circuit and the switching voltages can be controlled by changing the hysteresis resistors. This comparator was chosen after comparing and testing it with two other comparators as shown in Table 6.

**Figure 25.**
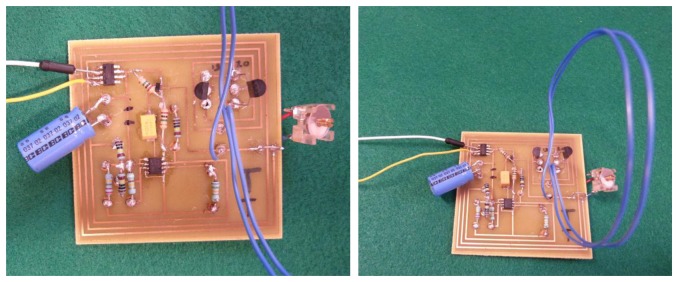
Layout of the power management unit and the capacitive sensor read-out circuitry (65 × 65 mm^2^).

**Figure 26.**
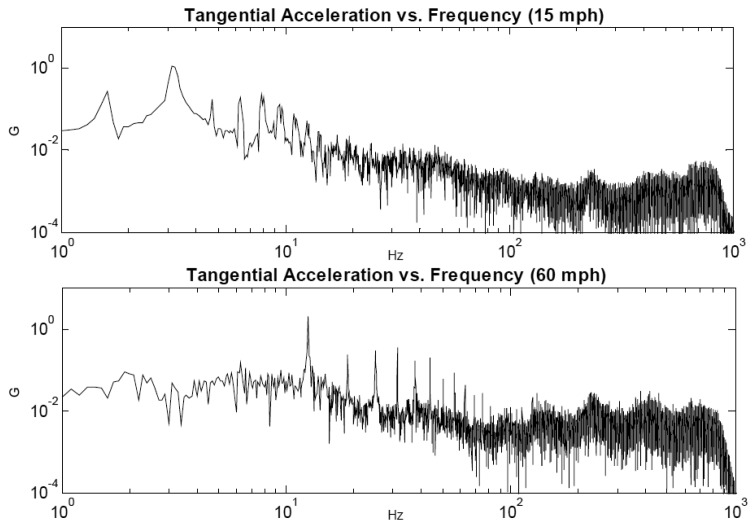
Tangential acceleration spectrum at 15 and 60 mph (24 and 96 km/h) (reproduced from [16] with permission).

**Figure 27.**
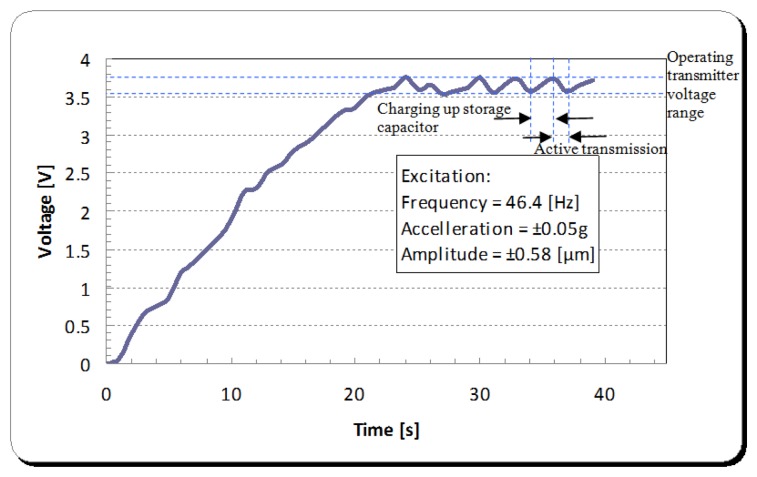
Voltage built up in the storage capacitor of the energy harvester and read-out circuitry assembly.

**Figure 28.**
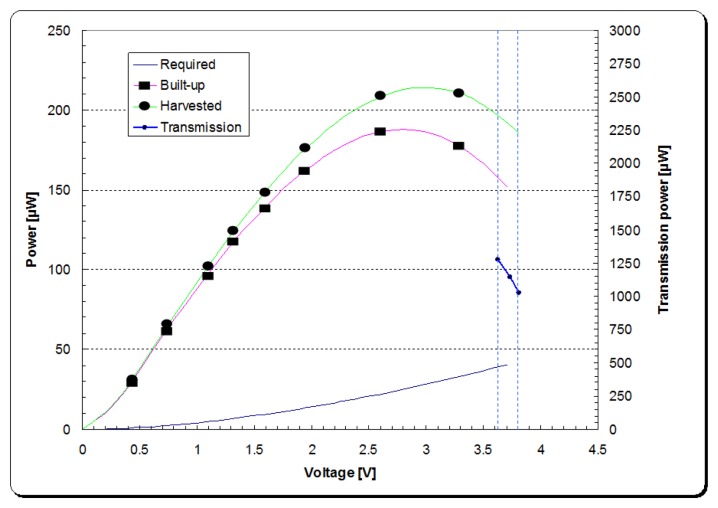
Power balance of the energy harvester and read-out circuitry assembly.

The PCB layout of the integrated power management unit with the λ oscillator/transmitter is shown in Figure 25. Given the tangential acceleration component inside a rolling pneumatic tyre shown in Figure 26 [16], the developed energy harvester was tested at a low level of acceleration excitation at resonance. The available shaker was capable of delivering as low as ±0.05 g of excitation acceleration, which is within the excitation range occurring in a rolling wheel as presented in Figure 26, and thereby the system was tested at that acceleration level to approach vibration conditions within a rolling pneumatic tyre as much as possible. Acceleration data were gathered by affixing an accelerometer on an R13 rim inside tyre cavity. The transmitted signal is a frequency signal, meaning that the RF frequency of the received signal is driven by the vale of the capacitance in the capacitive sensor. The conducted results at steady state charging-discharging cycle (Figures 27 and 28) showed an average sampling rate of approximately 0.166% (9.95 sample/min). The switching voltage range, bordered using two blue dashed lines in Figures 27 and 28, was chosen to ensure a reasonable balance between the transmitter/oscillator power consumption and the amount of the generated power by the energy harvester.

## 6. Conclusions

A study of a vibration-based cantilever energy harvester is presented in this article. Three vibration-based energy harvester designs are compared theoretically, numerically and experimentally to determine the influence of the harvester geometry on the overall device power output and efficiency. The device, of which the highest power output was achieved, was employed as the power supply of the λ-diode oscillator/transmitter designed by the author under vibration conditions close to those existing in a rolling tyre, to determine its feasibility of powering a TPMS.

The experimental results show a successful attempt to boost the power output level, in a thin air-spaced cantilever beam for energy within the same space envelope, and virtually quadruple the energy harvester efficiency when the same excitation amplitude is applied.

However, in this type of energy harvesting, assuming no contribution from resonance or harmonics, the output power of such a system is inversely proportional with the square of the excitation frequency [16], and usually these systems require adding a proof mass to enhance their efficiency. For these two reasons, and for powering a TPMS, an alternative energy harvesting system is required, e.g., a multi-resonance vibration energy harvester or a direct strain energy harvester that can extract energy from cyclic tyre deformation. Having the energy harvester installed inside tyre cavity, a robust packaging is essential. Ideally, the energy harvester needs to be as close and compact as possible with the TPMS electronics and therefore containing the energy harvester inside the TPMS casing is desirable.

## Figures and Tables

**Table 1.** Summary of published research for energy harvesters designed potentially for self powered TPMS.

**Energy Harvesting Mechanism**	**Energy Harvesting Technique**	**Size**	**Power Output**	**Output Voltage**	**Location within the Tyre**	**Source**
Vibration	Electromagnetic	5 mm diameter × 5 mm high magnet, no more specifications are given	0.054 mW at 60 km/h	1.5 VAC at 60 km/h	Attached onto the inner surface of the tyre belt	Tornincasa *et al.*, 2012 [24]
Vibration	Piezoelectric	31.8 × 3.2 × 0.66 mm^3^	0.78 μW at 50 km/h 2.99 μW at 80 km/h	2–3 V at 50 km/h 5–10 V at 80 km/h	Attached to the tyre wall from the outside in the tangential direction at 16 cm distance from the wheel centre	Pinna, 2010 [27]
Vibration	Piezoelectric	55.4 × 15.2 × 1.2 mm^3^	100.4 μW at resonance frequency (47.6 Hz)	6 VAC at resonance frequency (47.6 Hz)	-	Chen and Pan, 2011 [22]
Vibration	Piezoelectric	Some 10 mm^2^ in area × 80 μm in thickness	5.5 μW at resonance (11 kHz)	3.7 VAC at resonance (11 kHz)	Either on the rim or in the inner liner of the tyre	Frey, 2011 [28]
Vibration	Electromagnetic	30 × 30 × 11.7 mm^3^	0.144 mW at 50 km/h at an acceleration of 6g, 0.4 mW at 150 km/h at an acceleration of 15g	120 mV at 50 km/h at an acceleration of 6g, 200 mV at 150 km/h at an acceleration of 15g	-	Hatipoglu and Urey, 2009 [20]
Vibration	Piezoelectric	A diameter of 10.4 mm × 1.4 mm thickness	Peak power = 80 μW at 80 km/h, average power of 40 μW over 30–180 km/h speed range	Maximum of 40 V (open circuit) conditions are not specified	The sensor module mounted at the inner tread area	Keck, 2007 [9]
Vibration	Piezoelectric	Not specified	Averaged power of 0.38 mW	Maximum 12.3 V at resonance, 125.8 Hz (open circuit)	-	Liji WU *et al.*, 2009 [10]
Vibration	Piezoelectric	15 × 6 × 46 mm^3^	47 μW at approximately 80 km/h at resonance	>5 VAC but not specified	The vibration energy harvesting device was mounted on the wheel up-side-down to make sure the PZT operates in compression mode	Zheng *et al.*, 2009 [11]
Vibration	Piezoelectric	(≈10) × 20 × 20 mm^3^	123 μW at 16.2 Hz 60 μW at 6.2 Hz	(21–25) V_p-p_ over the frequency range (4–16) Hz in which the system almost remains at resonance	The device is mounted at optimal radius of 7.5 mm from the centre of rotation	Lei Gu, and Livermore, 2010 [29]
Vibration	Piezoelectric	Not clear	Average power of 10 μW over the frequency range (10–22) Hz under 1 g acceleration of excitation	Average voltage ≈ 14V_p-p_ across a 6.1 MΩ resistive load	The harvester mounted on the rim inside the tyre cavity	Tang *et al.*, 2012 [18]
Vibration	Electromagnetic	≈ 2.5 cm in diameter, thickness is not specified	Average power of 349 μW at 400 rpm across a 330 Ω resistive load	≈ 0.33 V_rms_ at 400 rpm across a 330 Ω resistive load	The weighted pendulum combined with magnets and coils was mounted on a rotation plate driven by an ac servo motor to simulate the device oscillation.	Wang *et al.*, 2012 [19]

**Table 2.** The governing equations of the vibration energy harvester.

**Parameter Description**		**Symbol**	**Units**	**Formulae**
Pure bending	Stiffness	*k*_P_	N/m	$k_{P}=\frac{4R_{P}}{l{\left(l+l_{pm}\right)}^{2}}$
	Rigidity	*R*_P_	N m^2^	$R_{P}=E_{1}(I_{1}+A_{1}{d_{1}}^{2})+E_{2}(I_{2}+A_{2}{d_{2}}^{2})$
S-shape bending	Stiffness	*k*_S_	N/m	$k_{S}=\frac{12R_{S}}{l^{3}}$
	Rigidity	*R*_S_	N m^2^	$R_{S}=E_{1}I_{1}+E_{2}I_{2}$
Effective stiffness		*k*_E_	N/m	$k_{E}=\frac{k_{P}k_{S}}{k_{P}+k_{S}}$
Resonance frequency		*f*_0_	Hz	$f_{0}=\frac{1}{2\pi }\sqrt{\frac{k_{E}}{m}}$
Normal strain acting along the x-axis in the DPT element		(ε_x_)_avg_	-	${\left({ɛ}_{x}\right)}_{\text{avg}}=\frac{ma(l+l_{pm})d_{2}}{2R_{P}}$

**Table 3.** Design parameters of the three cantilever-DPT assemblies.

**Parameter**		**Units**	**Design A**	**Design B**	**Design C**
Cantilever dimensions					

*w*_1_	width	m	3.50 × 10^−2^	3.50 × 10^−2^	1.75 × 10^−2^
*t*_1_	thickness	m	8.00 × 10^−4^	8.00 × 10^−4^	8.00 × 10^−4^
*l*_1_	length	m	4.50 × 10^−2^	4.50 × 10^−2^	4.50 × 10^−2^
*m_c_*	mass	kg	1.42 × 10^−2^	1.42 × 10^−2^	9.33 × 10^−3^

DPT element dimensions					

*w*_2_	width	m	3.50 × 10^−2^	3.50 × 10^−2^	3.50 × 10^−2^
*t*_2_	thickness	m	4.00 × 10^−4^	4.00 × 10^−4^	4.00 × 10^−4^
*l*_2_	length	m	4.50 × 10^−2^	4.50 × 10^−2^	4.50 × 10^−2^
*m_dpt_*	Mass	kg	2.10 × 10^−3^	2.10 × 10^−3^	2.10 × 10^−3^

Proof mass					

m_pm_*	mass	kg	5.14 × 10^−2^	5.14 × 10^−2^	5.14 × 10^−2^
*l*_pm_	length	m	2.00 × 10^−2^	2.00 × 10^−2^	2.00 × 10^−2^

Cantilever properties					

*E*_1_	modulus of elasticity	Pa	2.00 × 10^11^	2.00 × 10^11^	2.00 × 10^11^
*I*_1_	second moment of area	m	1.49 × 10^−12^	1.49 × 10^−12^	7.47 × 10^−13^
*A*_1_	x-area	m^2^	2.80 × 10^−5^	2.80 × 10^−5^	1.40 × 10^−5^
*d*_1_		m	1.04 × 10^−5^	1.04 × 10^−5^	2.42 × 10^−5^

DPT element properties					

*E*_2_	Modulus of elasticity	Pa	4.40 × 10^9^	4.40 × 10^9^	4.40 × 10^9^
*I*_2_	Second moment of area	m	1.87 × 10^−13^	1.87 × 10^−13^	1.87 × 10^−13^
*A*_2_	x-area	m^2^	1.40 × 10^−5^	1.40 × 10^−5^	1.40 × 10^−5^
*d*_2_		m	5.89 × 10^−4^	5.90 × 10^−4^	1.38 × 10^−3^

Centroid from the fixed end					

*x̅*		m	5.37 × 10^−2^	5.37 × 10^−2^	5.48 × 10^−2^
*m*		kg	6.77 × 10^−2^	6.77 × 10^−2^	6.28 × 10^−2^

Cantilever-DPT assembly rigidity					

*D*		m	6.00 × 10^−4^	1.40 × 10^−3^	1.40 × 10^−3^
*R_p_*		N.m^2^	3.22 × 10^−1^	4.19 × 10^−1^	2.69 × 10^−1^
*R*_s_		N.m^2^	2.99 × 10^−1^	2.99 × 10^−1^	1.50 × 10^−1^

Cantilever-DPT assembly stiffness					

*k*_S_		N/m	3.94 × 10^4^	3.94 × 10^4^	1.98 × 10^4^
*k*_p_		N/m	6.76 × 10^3^	8.82 × 10^3^	5.66 × 10^3^
*k*_E_		N/m	5.77 × 10^3^	7.21 × 10^3^	4.40 × 10^3^

Resonance frequency					

*f*_0_		Hz	53.4	59.6	46.6

Normal strain in the DPT element under 0.5 g acceleration of excitation (*a*)					

*a*	acceleration	m/s^2^	4.905	4.905	4.905
(*ε*_x_)_avg_		με	15.0181	26.8651	41.145

* The mass used in the calculations is the sum of the proof mass and the 1.8 × 35 × 10 mm^3^ (4.37 × 10^−3^ kg) portion at the end of each of the three cantilevers.

**Table 4.** Summary of the FEA simulation results.

**Parameter**	**Description**	**Units**	**Design A**	**Design B**	**Design C**
*V*_avg_	the average voltage generated by the DPT	V	4.96	5	7.149
*V*_max_	maximum voltage within the DPT	V	6.832	7.155	10.438
*F*	applied force on the cantilever-DPT assembly	N	1.4	1.4	1.4
*u*_end_	deflection of the free end	mm	0.134	0.0515	0.0891
*u*_cg_/*u*_end_	deflection at the centre of gravity of the cantilever-DPT assembly relative to the deflection of the free end	mm	0.708	0.708	0.783
*k*	stiffness	kN/m	10.447	27.184	15.713
*ε*_avg_	normal strain in the DPT element in the x direction	με	33.662	34.024	48.59
*f*_0_	resonance frequency	Hz	48.13	64.58	50.255

**Table 5.** Summary of the test results for the three vibration based energy harvester designs.

**Common Variables**			**Design**					
Parameter	Description	Units	A		B		C	
*f*_0_	Natural frequency	Hz	5.98 × 10^1^		5.85 × 10^1^		4.76 × 10^1^	
*τ_d_*	Oscillation period	s	1.67 × 10^−2^		1.71 × 10^−2^		2.10 × 10^−2^	
*c*	Damping coefficient	N s/m	4.45 × 10^−1^		4.75 × 10^−1^		4.07 × 10^−1^	

**Excitation Variables**			**±0.5g Acceleration of Excitation**			**±40 μm Amplitude of Excitation**		
	
			**A**	**B**	**C**	**A**	**B**	**C**

*z*_0_	Excitation amplitude	m	3.49 × 10^−5^	3.63 × 10^−5^	5.48 × 10^−5^	4.00 × 10^−5^	4.00 × 10^−5^	4.00 × 10^−5^
${\ddot{z}}_{0}$	Peak excitation acceleration	m/s^2^	4.91 × 10^0^	4.91 × 10^0^	4.91 × 10^0^	5.63 × 10^0^	5.40 × 10^0^	3.58 × 10^0^
*H*_inp_	Energy input per cycle	μJ/Cycle	1.19 × 10^5^	8.92 × 10^4^	7.91 × 10^4^	1.56 × 10^5^	1.08 × 10^5^	4.22 × 10^4^
(*H*_out_)_avg_	Average energy output per cycle	μJ/Cycle	1.48 × 10^1^	1.90 × 10^1^	2.79 × 10^1^	2.04 × 10^1^	2.16 × 10^1^	1.81 × 10^1^
(*H*_out_)_max_	Maximum energy output per cycle	μJ/Cycle	2.24 × 10^1^	2.87 × 10^1^	3.99 × 10^1^	3.08 × 10^1^	3.30 × 10^1^	2.56 × 10^1^
*P*_avg_	Average power generation	mW	8.84 × 10^−1^	1.11 × 10^0^	1.33 × 10^0^	1.22 × 10^0^	1.26 × 10^0^	8.60 × 10^−1^
*P*_max_	Maximum power generation	mW	1.34 × 10^0^	1.68 × 10^0^	1.90 × 10^0^	1.84 × 10^0^	1.93 × 10^0^	1.22 × 10^0^
*P*_dns_	Volume power density	mW/cm^3^	7.93 × 10^−2^	9.94 × 10^−2^	1.12 × 10^−1^	1.09 × 10^−1^	1.14 × 10^−1^	7.22 × 10^−2^
*η*	Total efficiency	%	1.89 × 10^−2^	3.22 × 10^−2^	5.05 × 10^−2^	1.44 × 10^−2^	2.90 × 10^−2^	6.07 × 10^−2^

**Table 6.** A comparison between three selected comparators.

Part	ICL7665S	MAX6763/MAX6764	MAX971/MAX981

Properties			
Supply Current/μA	2.55	23	4
Operating Temperature Range/°C	0 to 70	−40 to 125	0 to 120
No. of required resistors	7	3 (a relay and NPN transistor is required)	5
Chip dimensions/mm	5 × 6 × 1.75 (10.16 × 7.11 × 5.33)	3 × 3 × 1.45 + relay & transistor	5.03 × 4.78 × 1.10
